# Supporting youth mental health with arts-based strategies: a global perspective

**DOI:** 10.1186/s12916-023-03226-6

**Published:** 2024-01-03

**Authors:** Tasha L. Golden, Richard W. Ordway, Susan Magsamen, Aanchal Mohanty, Yifan Chen, T. W. Cherry Ng

**Affiliations:** 1 International Arts + Mind Lab, Johns Hopkins University School of Medicine, 5801 Smith Ave, Ste #110, Baltimore, MD 21209 USA; 2 Department of Biology and Doctors, Kienle Center for Humanistic Medicine, Penn State University, University Park, PA 16802 USA

**Keywords:** Arts and culture, Youth mental health, Community care

## Abstract

The devastating impact of youth mental health concerns is increasingly evident on a global scale. This crisis calls for innovative solutions that are sufficiently accessible, scalable, and cost-effective to support diverse communities around the world. One such solution involves engagement in the arts: incorporating and building upon existing local resources and cultural practices to bolster youth mental health. In this article, we describe the global youth mental health crisis and note major gaps in the knowledge and resources needed to address it. We then discuss the potential for arts- and culture-based strategies to help meet this challenge, review the mounting evidence regarding art’s ability to support mental health, and call for action to undertake critical research and its translation into accessible community practices. Four steps are suggested: (1) elevate and prioritize youth voice, (2) develop core outcome measures, (3) identify and analyze successful models around the globe, and (4) generate clear funding pathways for research and translational efforts. Worldwide implementation of arts- and culture-based strategies to address youth mental health will provide critical resources to support the health, wellbeing and flourishing of countless youth across the globe.

## Background

Globally, mental health and substance use disorders are the leading cause of disability in children and youth [1] and suicide is a leading cause of death among people aged 10–24 years [2, 3–5]. Youth ages 10–19 make up 16% of the world’s population (1.2 billion individuals), and 14% of this population experiences mental health issues, with emotional and behavioral disorders being most common [2, 6]. Given that adolescents and young adults are in formative developmental periods in which they are uniquely sensitive to their environments, physical and socioemotional changes [7]— as well as exposure to poverty, violence, or abuse—can increase susceptibility to mental health issues [8–11]. In addition, greater risks are experienced by youth who are neurodivergent or have learning disabilities; youth with neurological disorders; youth who are pregnant or forced into early marriages; youth exposed to stigma; youth from minority racial, ethnic, religious, or other groups that face exclusion or prejudice; and/or youth lacking equitable access to quality healthcare [2]. Mental health challenges among youth are on the rise in many parts of the world and appear to have been exacerbated by the COVID-19 pandemic [5, 12]; the devastating impacts of these challenges are broad. Fifty percent of long-term mental health problems begin by age 14 and 75% begin by age 24 [13, 14]. Emerging mental disorders during one's youth are associated with sustained mental health problems and lower overall functioning in adulthood [15, 16]. In economic terms, the global cost of these conditions is estimated to be $16.1 trillion between 2010 and 2030 [17].

Despite the extent of this crisis, major gaps persist in the resources needed to investigate and address youth mental health, including shortages of mental health professionals [18–20]. For example, 85% of children worldwide live in low- and middle-income countries (LMICs), yet youth mental health concerns are often overlooked in LMICs and in underrepresented communities within high-income countries [1, 21]. The resulting lack of prevalence data precludes effective analysis of youth mental health needs and the equitable distribution of resources. In addition, youth around the world often face barriers in access to mental health treatment, including primary and secondary care, evidence-based treatment, and engagement with the mental healthcare infrastructure [22–27].

In short, the extent of youth mental health concerns, and the limited resources and personnel currently available to address them, pose a significant challenge to protecting and promoting youth health. The daunting nature of this challenge demands that resources be directed toward a wide range of innovative, age-appropriate, and culturally sustaining solutions. By intervening early with evidence-based youth programs and services, we have the potential to strengthen youths’ mental health [28, 29] and to reduce mental health challenges in adulthood.

## The value of arts- and culture-based assets

A comprehensive analysis of potential evidence-based resources and services to improve youth mental health is not the aim of this article; rather, we aim specifically to highlight the value of the arts- and culture-based assets and practices as sources of community-based support and adjunctive care. Social determinants of health—community and contextual factors that determine one’s ability to be healthy—have been shown to have greater influence on health than clinical care [30, 31], affecting one’s safety, education and employment, opportunities and resources, and both physical and psychological well-being [32–34]. As a result, serious efforts to address youth mental health must consider the role of community and contextual factors in bolstering and sustaining health. Notably, given the health benefits of arts, culture, and nature, access to these resources is now understood as a social determinant of health [35], and engagement in the arts is increasingly recognized as a powerful and potentially scalable way to promote mental health and wellbeing. For example, when arts interventions have been used with developmentally diverse populations, they have generated a wide range of positive outcomes and improvement in the overall quality of life [36, 37] and have been shown to promote togetherness [38]. In addition, across culturally diverse populations of youth, engagement in local arts and cultural practices was found to bolster mental health by promoting an enhanced sense of community, self-expression, and cultural identity, which are protective factors against mental health symptoms and distress prompted by discrimination [39–41]. Indeed, approaches that are grounded in local arts and culture practices can be distinguished from other interventions by their potential to offer relevance to a wide range of diverse communities in various settings, and by their relative cost-effectiveness [36]. Beyond direct health benefits, the arts can also improve communication and education regarding stigmatized topics such as mental illness by generating messages that are culturally relevant, memorable, and actionable [42]. Finally, the World Health Organization's (WHO) definition of health includes not only the absence of disease but also the cultivation of well-being (e.g., meaning, connection, flourishing) [43]. It is therefore critical that responses to the mental health crisis incorporate the positive impacts of arts-based experiences and opportunities on youths’ well-being and ability to flourish [44–47].

Mechanisms for the effectiveness of arts-based programs in supporting mental health have been explored by numerous studies across multiple fields and disciplines. For example, psychological and psychophysiological assessments indicate that arts interventions increase emotional and cognitive arousal [48], promote self-concept and stress resilience [49], increase sleep quality, and improve overall well-being [37]. In neuroscience, imaging technology has demonstrated neurobiological changes due to arts-based stimuli [50]. In addition, aesthetic evaluation has been shown to activate neural nuclei involved in the mesocorticolimbic reward circuit [51], and increased activation and functional interaction within the system is associated with high resilience in those with high adversity experiences [52]. Engagement in creative tasks also activates the hippocampal cortex in the limbic system–increasing alpha and theta brain waves associated with relaxed states, meditation, and memory retrieval [53–55]. Moreover, in clinical settings, various art forms have been used to treat people with developmental disorders or traumatic brain injury, and have been shown to have positive biological and psychological effects [56, 57].

At community- and population-levels, studies have demonstrated the capacity for arts-based strategies to raise mental health awareness [58, 59], address stigma [58, 60–63], treat mental health symptoms [58, 64], and promote recovery, coping, and resilience [58, 65–71]. Public health research has also identified the role of arts and culture in generating social connections and fostering dialogue around difficult issues [40, 72, 73]. Finally, creative arts have been shown to offer trauma-informed and culturally responsive ways for researchers to learn about difficult experiences among youth [61].

## Call to action

Despite the mounting evidence regarding their relevance and effectiveness, arts and culture strategies are not yet standard considerations in efforts to support youth mental health. Further research and evaluation are needed to understand the effects of arts-based practices on youth mental health, the mechanisms driving these effects, and potential applications. Most obviously, further research is needed to identify youths’ mental health needs and experiences, as well as their arts and cultural interests and preferences, across various cultures, nations, geographies, religions, neurotypes, education levels, socioeconomic status, etc. In addition, there is a pressing need to document *existing* arts- and culture-based practices, programs, resources, and services that are of relevance and interest to varied youth populations–and thus have the potential to serve as mental health supports. Such programs as The Simple Good, Healing Arts at S.P.Y., and BalletRox may already be providing support for youth mental health but lack formal evaluation studies to measure and document their impact(s). It is also likely that some youth arts- and culture-based practices provide mental health support without explicitly aiming to do so. Consequently, research into current youth arts practices, existing community cultural assets, and successful youth cultural activities is likely to generate insight into youth mental health supports through an assets-based, culturally relevant, community-centric approach.

Beyond identifying promising existing practices to inform and bolster youth mental health research, there is also a need to translate existing research into the further development of culturally relevant practices. For example, accumulating findings regarding the mental health benefits of specific arts-based practices, expressive therapies, or formally evaluated arts programs should be translated into accessible practices for individuals and youth-serving organizations. They could also be applied via education and training to enhance existing youth programs or develop new ones.

## Next steps

The preceding research objectives may be pursued in myriad ways, and the authors do not wish to prescribe or circumscribe this process. That said, given the sheer breadth of potential approaches, we have identified four suggestions to guide effective study into the value of arts- and culture-based strategies in supporting youth mental health. Each of these steps is detailed below.

First, we emphasize the importance of elevating youth voice and leadership in all efforts to address youth mental health and to understand and evaluate links between arts- and culture-based practices and mental health outcomes. When youth voice is elicited in rigorous ways and combined with insights from expert researchers and practitioners, youths’ input and lived experience will provide a strong foundation from which to bolster youth mental health on a global scale.

Second, we recommend that arts- and culture-based programs identify robust, transparent processes for assessing their effects on youth mental health. While evidence points to the clear capacity and promise for arts and culture to promote health and reduce health inequities, researchers have called for rigorous, systematic, and transparent methods to guide practice, policy, and future progress [74–77]. It is thus critical that each individual study or series of studies explain the outcomes they choose to measure as well as any other criteria used to assess the effectiveness of a given program or practice in protecting, promoting, or treating youth mental health. Over time, the synthesis of such studies will facilitate comparison, objective evaluation, and optimization of program practices.

Third, given differential effects across cultures, regions, and backgrounds, a fully comprehensive global examination of youths’ needs, experiences, practices, and preferences may be time- and cost-prohibitive. We thus recommend identifying a representative group of programs or practices that have been implemented with a wide range of youth populations, including programs that appear to be noteworthy or successful based on criteria developed by experts and youth together (i.e., long-running, high engagement, integrated with health systems, integrated with school systems, etc.). A group of such model programs could then be analyzed in-depth. An important element of this type of analysis is the collaboration and integration of research groups representing distinct cultural and regional backgrounds as well as complementary fields of expertise.

Finally, as a concrete means of advancing these efforts, we call upon funding bodies to create grant opportunities and requests for proposals related to this research. It should be recognized that examining and funding arts- and culture-based practices for youth mental health is not distinct from examinations of and funding for promising community-based practices, non-medical referrals, and social programs in general. Indeed, the integration of well-regarded, culturally relevant community practices into healthcare, education, and policymaking practices is a known means of bolstering participation, access, equity, and sustained effort [78, 79].

As Sonke and colleagues [62] have noted, every community has arts and culture assets that can and should be integrated into efforts to protect and promote community and population health. This is true of youth populations as well, and our ability to honor, tap into, and build upon youths’ arts and cultural practices can only benefit efforts to support and advance their health.

## Conclusions

The enormous challenges presented by the global youth mental health crisis demand solutions that are effective as well as sufficiently accessible, adaptable, and scalable to reach a wide range of populations. The documented success of arts- and culture-based strategies in promoting mental health, together with their potential to integrate with and expand upon existing cultural resources within communities, make them highly attractive tools to support youth mental health globally. This article has noted deficiencies in the collective understanding of the current global crisis, as well as in the essential resources needed to improve youth mental health. In response, it has recommended arts- and culture-based strategies that involve intensive research and its translation into actionable practice. With concerted efforts of this kind on a global scale, we will make meaningful progress in the fight to alleviate youth mental health concerns and promote the well-being and equity of youth around the world.

## Data Availability

Not applicable.

## Abbreviation

LMIC: Low and middle-income countries
